# Interpolation-Based Semantic Gate Extraction and Its Applications to QBF Preprocessing

**DOI:** 10.1007/978-3-030-53288-8_24

**Published:** 2020-06-13

**Authors:** Friedrich Slivovsky

**Affiliations:** 8 Microsoft Research Lab, Redmond, WA USA; 9 University of Southern California, Los Angeles, CA USA; TU Wien, Vienna, Austria

## Abstract

We present a new semantic gate extraction technique for propositional formulas based on interpolation. While known gate detection methods are incomplete and rely on pattern matching or simple semantic conditions, this approach can detect any definition entailed by an input formula.

As an application, we consider the problem of computing unique strategy functions from Quantified Boolean Formulas (QBFs) and Dependency Quantified Boolean Formulas (DQBFs). Experiments with a prototype implementation demonstrate that functions can be efficiently extracted from formulas in standard benchmark sets, and that many of these definitions remain undetected by syntactic gate detection.

We turn this into a preprocessing technique by substituting unique strategy functions for input variables and test solver performance on the resulting instances. Compared to syntactic gate detection, we see a significant increase in the number of solved QBF instances, as well as a modest increase for DQBF instances.

## Introduction

Due to the effectiveness of modern satisfiability (SAT) solvers [20], propositional logic has become the language of choice for encoding hard combinatorial problems arising in areas such as electronic design automation [50] and AI planning. Since many of these problems are hard for levels of the polynomial hierarchy beyond NP, their propositional encodings can be exponentially larger than their original descriptions. This imposes a limit on the problem instances that can be feasibly solved even with extremely efficient SAT solvers, and has prompted research on decision procedures for more succinct logical formalisms such as Quantified Boolean Formulas (QBFs).

Quantified Boolean Formulas (QBFs) are propositional formulas combined with universal and existential quantification over truth values and offer much more succinct encodings of problems from domains such as planning and synthesis [12]. At the same time, QBF evaluation is PSPACE-complete, and in spite of substantial progress in solver technology, many practically relevant instances remain hard to solve.

In part, this hardness appears to be a matter of encoding. The most commonly used format for QBFs is Prenex Conjunctive Normal Form (PCNF). A PCNF formula consists of a quantifier prefix and a matrix in conjunctive normal form. As in the case of propositional logic, any QBF can be converted to PCNF with linear overhead but this transformation is known to adversely affect solver performance [1]. This appears to be due to two issues: First, conversion to CNF causes a bias towards reasoning about unsatisfiability while making it difficult to reason about solutions, violating the inherent duality of QBF solving. Second, prenexing introduces spurious variable dependencies that needlessly constrain solvers [5, 40]. In light of these issues, researchers have introduced two new formats for representing non-CNF (and even non-prenex) QBFs in the QCIR [30] and QAIGER standards, and solvers supporting these standards have been developed. When only a PCNF encoding is available, *gate extraction* techniques can be used to (re)construct a non-CNF QBF [21]. *Syntactic* gate extraction relies on the detection of patterns of clauses and auxiliary variables introduced when converting a propositional formula to CNF [16]. The corresponding algorithms are fast but incomplete and can only detect definitions from a pre-defined library of gates.

In this paper, we introduce a new semantic gate extraction technique based on SAT solving and interpolation. In contrast to known approaches, this method is complete: a definition $\psi$ of a variable *x* can be extracted from a propositional formula $\varphi$ whenever the equivalence $x \equiv \psi$ is entailed by $\varphi$. We obtain this result as a generalization of recent work that leverages definability for propositional model counting [25, 33]. Owing to a result known as Padoa’s Theorem, determining whether a variable *x* is definable in terms of *X* is in coNP and can be decided by a SAT call [33]. We show that a definition $\psi$ of *x* in terms of *X* can be obtained as an *interpolant* of the formula passed to the SAT solver (Theorem 2). For SAT solvers that use a proof system with feasible interpolation—in particular, CDCL solvers that generate resolution proofs [32]—this means a definition can be efficiently extracted from a proof of definability.

We apply this new gate extraction technique to identify unique strategy functions of QBFs and Dependency QBFs. In a controller synthesis setting, a variable with a unique strategy function corresponds to a control signal with a unique (as a Boolean function) implementation. We can add such an implementation to the specification without affecting the remaining control signals.

Experiments with a prototype show that definitions can be efficiently computed for formulas from standard QBF benchmark sets, and that for many instances a large fraction of variables have unique strategy functions that cannot be identified by syntactic gate detection. We further test the performance of solvers on instances obtained by replacing input variables with their definitions. For 2QBF formulas and PCNF formulas, this significantly increases the number of instances solved by some systems compared to purely syntactic gate extraction. Our experiments further show that semantic gate detection is orthogonal to techniques implemented in state-of-the-art preprocessors.

Semantic gate detection is efficient and conceptually simple. By definition, it preserves logical equivalence and is compatible with strategy extraction. As such, we believe it is an essential addition to the state of the art in preprocessing (D)QBF.

## Preliminaries

We assume a countably infinite set *V* of propositional *variables* and consider *propositional formulas* constructed from *V* using the connectives $\lnot$ (negation), $\wedge$ (conjunction), $\vee$ (disjunction), $\rightarrow$ (implication), and $\leftrightarrow$ (the biconditional). For a propositional formula $\varphi$, we write $var (\varphi )$ to denote the set of variables occurring in $\varphi$. A *literal* is a variable *v* or a negated variable $\lnot v$. A *clause* is a finite disjunction of literals. A clause is *tautological* if it contains both *v* and $\lnot v$ for some variable *v*. A propositional formula is in *conjunctive normal form (CNF)* if it is a finite conjunction of non-tautological clauses. An *assignment* of a subset $X \subseteq V$ of variables is a function that maps *X* to the set $\{0, 1\}$ of truth values. For a set *X* of variables we let [*X*] denote the set of assignments of *X*. Two assignments $\sigma : X \rightarrow \{0, 1\}$ and $\tau : Y \rightarrow \{0, 1\}$ *agree* on a subset $W \subseteq X \cap Y$ of their common domain if $\sigma (w) = \tau (w)$ for each $w \in W$. For two assignments $\sigma : X \rightarrow \{0, 1\}$ and $\tau : Y \rightarrow \{0, 1\}$ that agree on the entire intersection of their domains we define the combined assignment $\sigma \cup \tau : X \cup Y \rightarrow \{0, 1\}$ as $(\sigma \cup \tau )(v) = \sigma (v)$ if $v \in X$ and $(\sigma \cup \tau )(v) = \tau (v)$ otherwise.

For a propositional formula $\varphi$ and an assignment $\tau : X \rightarrow \{0, 1\}$ with $var (\varphi ) \subseteq X$, we let $\varphi [\tau ]$ denote the truth value obtained by evaluating $\varphi$ under $\tau$. The formula $\varphi$ is *satisfied* by $\tau$ if $\varphi [\tau ] = 1$. In this case we call $\tau$ a *satisfying assignment* of $\varphi$. Otherwise, if $\varphi [\tau ] = 0$, formula $\varphi$ is *falsified* by $\tau$. A formula is *satisfiable* if it has a satisfiable assignment, otherwise it is *unsatisfiable*. A formula $\varphi$ *implies* a formula $\psi$ if $\varphi \wedge \lnot \psi$ is unsatisfiable.

We consider Quantified Boolean Formulas (QBFs) in *Prenex Normal Form (PNF)*. A QBF $\varPhi = \mathcal {Q}.\varphi$ in PNF consists of a *quantifier prefix* $\mathcal {Q}$ and a propositional formula $\varphi$, called the *matrix* of $\varPhi$. The quantifier prefix is a sequence $Q_1x_1\dots Q_nx_n$ where $Q_i \in \{\forall , \exists \}$ and the $x_i$ are pairwise distinct variables for $1 \le i \le n$. The quantifier prefix defines an ordering $<_{\varPhi }$ on its variables as $x_i <_{\varPhi } x_j$ for $1 \le i < j \le n$. We assume that QBFs do not contain free variables and every variable in the quantifier prefix appears in the matrix, formally $\{x_1, \dots , x_n\} = var (\varphi )$. Accordingly, we write $var (\varPhi ) = var (\varphi )$ for the set of variables appearing in the QBF $\varPhi$. We further assume that every variable of $\varPhi$ occurs exactly once in its quantified prefix. The set of *existential* variables of $\varPhi$ is $var _{\exists }(\varPhi ) = \{\,x_i \;{|}\;1 \le i \le n, Q_i = \exists \,\}$, and the set of *universal* variables of $\varPhi$ is $var _{\forall }(\varPhi ) = \{\,x_i \;{|}\;1 \le i \le n, Q_i = \forall \,\}$. For a variable $x \in var (\varPhi )$, we let $type _{\varPhi }(x) = Q$ if $x \in var _Q(\varPhi )$, for $Q \in \{\forall , \exists \}$, omitting $\varPhi$ from the subscript if the QBF is understood.

Let $$\varPhi $$ a QBF and let $$x \in var (\varPhi )$$ be one of its variables with $$ type (x) = Q$$. A *strategy function* for *x* is a function $$f:[ var (\varPhi ) \setminus var _{Q}(\varPhi )] \rightarrow \{0, 1\}$$ such that $$f(\tau ) = f(\tau ')$$ for any two assignments $$\tau $$ and $$\tau '$$ that agree on variables in $$\{\,v \in var (\varPhi ) \setminus var _Q(\varPhi ) \;{|}\;v <_{\varPhi } x \,\}$$.^1^ Given an indexed family $$F = \{f_x\}_{x \in X}$$ of strategy functions such that $$X \subseteq var _Q(\varPhi )$$ for $$Q \in \{\forall , \exists \}$$, the *response* of *F* to an assignment $$\tau : ( var (\varPhi ) \setminus var _Q(\varPhi )) \rightarrow \{0, 1\}$$ is the assignment $$F(\tau ): X \rightarrow \{0, 1\}$$ given by $$F(\tau )(x) = f_x(\tau )$$. An *existential winning strategy* (for $$\varPhi $$) is a family $$F = \{f_u\}_{u \in var _{\exists }(\varPhi )}$$ of strategy functions such that, for any universal assignment $$\tau : var _{\forall }(\varPhi ) \rightarrow \{0, 1\}$$, the assignment $$\tau \cup F(\tau )$$ satisfies the matrix of $$\varPhi $$. Dually, a *universal winning strategy* (for $$\varPhi $$) is a family $$F = \{f_u\}_{u \in var _{\forall }(\varPhi )}$$ of strategy functions such that, for any existential assignment $$\sigma : var _{\exists }(\varPhi ) \rightarrow \{0, 1\}$$, the assignment $$\sigma \cup F(\sigma )$$ falsifies the matrix. A QBF $$\varPhi $$ is *true* if there is an existential winning strategy for $$\varPhi $$, and *false* if there exists a universal winning strategy for $$\varPhi $$.

## Semantic Gate Extraction by Interpolation

This work builds on an application of propositional *definability* to the model counting problem [33]. We begin by recalling two basic concepts.

### Definition 1

Let $\varphi$ be a formula, let *X* be a subset of its variables, and let *x* be a variable. Variable *x* is *defined* in terms of *X* in $\varphi$ if $\sigma (x) = \tau (x)$ for any two satisfying assignments $\sigma$ and $\tau$ of $\varphi$ that agree on *X*. A *definition* of *x* by *X* in $\varphi$ is a formula $\psi$ with $var (\psi ) \subseteq X$ such that $\sigma (x) = \psi [\sigma ]$ for any satisfying assignment $\sigma$ of $\varphi$.

It is readily verified that there is a definition for every variable that is defined. Lagniez et al. [33] observe that the following result can be used to determine whether a variable is defined [34, 39].

### Theorem 1 (Padoa’s Theorem)

Let $\varphi$ be a formula and let $X \subseteq var (\varphi )$ be a subset of its variables. Let $\varphi '$ be the propositional formula obtained by replacing every variable $y \in var (\varphi ) \setminus X$ by a new variable $y'$. Let $x \in var (\varphi )$ be a variable. If $x \notin X$, then *x* is defined in $\varphi$ by *X* if, and only if, the formula $\varphi \wedge x \wedge \varphi ' \wedge \lnot x'$ is unsatisfiable.

For the purposes of preprocessing in model counting, it is sufficient to know *that* a variable *x* is defined by *X* in $\varphi$, and the above result shows that this can be decided by a SAT solver. It is not necessary to compute the corresponding definition, whose size is not polynomially bounded in the size of $\varphi$ under common assumptions in computational complexity [33].

While finding definitions is harder than deciding definability in theory, the difference virtually disappears in practice. Our main theoretical contribution, stated as Theorem 2 below, says that a definition can be obtained as an *interpolant* of the formula constructed in the statement of Padoa’s Theorem. Since interpolants can be efficiently (in linear time) generated from resolution proofs [22, 32], the distinction between detecting definability and computing definitions becomes moot when a CDCL SAT solver is used to decide (un)satisfiability: once it determines that the formula is unsatisfiable it has already (implicitly or explicitly) produced a proof from which a definition can be extracted at a small overhead.^2^

Before proving Theorem 2, we recall the definition of an interpolant following McMillan [36].

### Definition 2 (Interpolant)

Let $\psi$ and $\chi$ be an formulas such that $\psi \wedge \chi$ is unsatisfiable. An *interpolant* for $\psi$ and $\chi$ is a formula *I* such that

1. $$\psi $$ implies *I*,
2. $I \wedge \chi$ is unsatisfiable, and
3. *I* only refers to variables common to $\psi$ and $\chi$.

Craig’s Interpolation Theorem [9] states that every pair of jointly unsatisfiable propositional formulas have an interpolant.^3^ It remains to show that an interpolant for a formula witnessing definability in fact yields a definition.

### Lemma 1

Let $\varphi$ be a formula and let $X \subseteq var (\varphi )$ be a subset of its variables. Let $\varphi '$ be the formula obtained by replacing every variable $y \in var (\varphi ) \setminus X$ by a new variable $y'$. For any variable $x \in var (\varphi ) \setminus X$, an interpolant for $\varphi \wedge x$ and $\varphi ' \wedge \lnot x'$ is a definition of *x* by *X* in $\varphi$.

### Proof

Let *I* be an interpolant for $\varphi \wedge x$ and $\varphi ' \wedge \lnot x'$. By property  of Definition 2, *I* only refers to the common variables $var (\varphi \wedge x) \cap var (\varphi ' \wedge \lnot x') = X$ of these formulas. To see that *I* defines *x* in $\varphi$, consider a satisfying assignment $\sigma : var (\varphi ) \rightarrow \{0, 1\}$ of $\varphi$. If $\sigma (x) = 1$ then $\varphi \wedge x$ is satisfied by $\sigma$. The formula $\varphi \wedge x$ implies *I* by property , so $I[\sigma ] = 1$ as well. Otherwise, $\sigma (x) = 0$ and we can construct a satisfying assignment $\sigma '$ of $\varphi ' \wedge \lnot x'$ by setting $\sigma '(v) = \sigma (v)$ for $v \in X$ along with $\sigma '(v') = \sigma (v)$ for $v \in var (\varphi ) \setminus X$. By property , $I \wedge \varphi ' \wedge \lnot x'$ is unsatisfiable, so we must have $I[\sigma '] = I[\sigma ] = 0$.

### Theorem 2

Let $\varphi$ be a formula and let $X \subseteq var (\varphi )$ be a subset of its variables. Let $\varphi '$ be the formula obtained by replacing every variable $y \in var (\varphi ) \setminus X$ by a new variable $y'$. A variable $x \in var (\varphi ) \setminus X$ is defined in terms of *X* in $\varphi$ if, and only if, the formula $\varphi \wedge x \wedge \varphi ' \wedge \lnot x'$ is unsatisfiable, and a definition of *x* in terms of *X* can be obtained as an interpolant for $\varphi \wedge x$ and $\varphi ' \wedge \lnot x'$.

### Proof

By Theorem 1 variable $x \in var (\varphi ) \setminus X$ is defined in terms of *X* in $\varphi$ if, and only if, the formula $\varphi \wedge x \wedge \varphi ' \wedge \lnot x'$ is unsatisfiable. Craig’s Interpolation Theorem tells us that in this case there is an interpolant for $\varphi \wedge x$ and $\varphi ' \wedge \lnot x'$, which defines *x* in terms of *X* by Lemma 1.

## Extracting Unique QBF Strategy Functions

In this section, we show how Theorem 2 can be used to extract unique strategy functions of QBFs. We say that the Skolem (Herbrand) function of an existential (universal) variable *x* in a QBF is *unique* if it is the same in every existential (universal) winning strategy. In particular, if *x* is existentially (universally) quantified and the formula is false (true), then the strategy function of *x* is trivially unique (there is none). In other words, the strategy function of a variable *x* is unique if there is *at most one* such function for *x* that is part of a winning strategy. The following result states that propositional definability is a sufficient condition for uniqueness of a strategy function.

### Proposition 1

Let $\varPhi = Q_1 x_1 \dots Q_n x_n.\varphi$ be a QBF. If an existential (universal) variable $x_i$ is defined in terms of variables $X \subseteq \{\,x_j \;{|}\;1 \le j < i, Q_j \ne Q_i\,\}$ in $\varphi$ ($\lnot \varphi$) its Skolem (Herbrand) function is unique.

### Proof

We only consider the case where $x_i$ is an existential variable of $\varPhi$ (the case where $x_i$ is a universal variable is symmetric). Let $F = \{f_{x_j}\}_{x_j \in var _{\exists }(\varPhi )}$ and $G = \{g_{x_j}\}_{x_j \in var _{\exists }(\varPhi )}$ be existential winning strategies and $\tau : var _{\forall }(\varPhi ) \rightarrow \{0, 1\}$ an assignment to the universal variables. Since *F* and *G* are existential winning strategies both $\sigma _F = \tau \cup F(\tau )$ and $\sigma _G = \tau \cup G(\tau )$ must be satisfying assignments of $\varphi$. The assignments $\sigma _F$ and $\sigma _G$ agree on $X \subseteq var _{\forall }(\varPhi )$, so we must have $f_{x_i}(\tau ) = \sigma _F(x_i) = \sigma _G(x_i) = g_{x_i}(\tau )$ because $x_i$ is defined in terms of *X*. Since $\tau$ was chosen arbitrarily, this identity holds for every universal assignment, so the functions $f_{x_i}$ and $g_{x_i}$ coincide.

To see that definability is not a *necessary* condition for a strategy function to be unique, consider the following example.

### Example 1

Let $\varPhi = \forall x \exists y \forall z. (x \leftrightarrow y) \vee z$. The formula $\psi = x$ represents the unique existential winning strategy (set *y* to the same value as *x*). However, variable *y* is not defined in terms of *x*: the assignments $\{x, y, z\}$ and $\{x, \lnot y, z\}$ both satisfy the matrix and agree on *x*, but differ on *y*. Intuitively, the reason why the existential strategy function for *y* is unique in spite of *y* not being defined is that the universal player would never assign *z* true as required by one of the assignments witnessing non-definability.

### An Algorithm for Computing Unique Strategy Functions

We now describe an algorithm for computing unique strategy functions of a QBF based on Proposition 1. By using an interpolating SAT solver (ItpSatSolver) that supports both incremental solving and assumptions [22], we can extract definitions for variables of a given quantifier type (universal or existential) using a single solver instance. Pseudocode is shown as Algorithm 1 below.

Let $\varPhi = Q_1 x_1 \dots Q_n x_n.\varphi$ be a QBF and let $Q \in \{\forall , \exists \}$ be a quantifier type. Algorithm 1 first determines the leftmost variable $x_i$ in the prefix of $\varPhi$ that has quantifier type *Q* (line 3). The strategy function of any variable to the right of $x_i$ in the prefix (including $x_i$ itself) may use the variables to its left (*shared*), so we can begin by looking for definitions of $x_i$ in terms of *shared*. Towards constructing the formula for the corresponding unsatisfiability check according to Theorem 2, $\textsc {copy}(\varphi , X)$ returns a copy $\varphi '$ of the matrix $\varphi$ where each variable $x \in var (\varphi ) \setminus shared$ has been replaced by a fresh variable $x'$. Next (lines 9–14), we consider each variable $x_j$ with quantifier type *Q*—these are the variables we want to find definitions of—and introduce two fresh “selector” variables $s_i$ and $s_i'$, while adding clauses $(\lnot s_j \vee x_j)$ and $(\lnot s_j' \vee \lnot x_j')$ to $\varphi$ and $\varphi '$, respectively. These clauses allow us to represent $\varphi \wedge x_j \wedge \varphi ' \wedge \lnot x_j'$ by assuming literals $s_j$ and $s_j'$.^4^

After initializing the SAT solver, we consider the variables $x_1, \dots , x_n$ in the order of the quantifier prefix (lines 18–29). If variable $x_j$ has quantifier type *Q*, we want to check whether $x_j$ is defined in $\varphi$ in terms of oppositely quantified variables $X_j$ that precede it in the prefix (Proposition 1 tells us that in this case the strategy function of $x_j$ is unique). For the first such variable $x_j$, it is clear that the set of variables common to $\varphi$ and $\varphi '$ is precisely *X*. Unsatisfiability of $\varphi \wedge x_j \wedge \varphi ' \wedge \lnot x_j'$ is decided by calling the SAT solver under assumptions $\{s_j, s_j'\}$: the assumptions ensure that $x_j$ and $\lnot x_j'$ are set to true by propagation, and all remaining selector variables can be set to false so as to satisfy the clauses they occur in without interfering with the remaining clauses. If the solver determines unsatisfiability, an interpolant $I_j$ is computed (line 22), which by Theorem 2 corresponds to a definition of $x_j$, and adds the pair $(x_j, I_j)$ to a list of definitions. Otherwise, if $x_j$ has the quantifier type opposite to *Q*, the strategy function of any variable with quantifier type *Q* considered later may use $x_j$. Accordingly (lines 26–27), we add clauses $(x_j \vee \lnot x_j')$ and $(\lnot x_j \vee x_j')$ to $\varphi '$ through the incremental interface of the SAT solver. This has two effects: first, it enforces equivalence of $x_j$ and $x_j'$, and second, $x_j$ is added to the common vocabulary of $\varphi$ and $\varphi '$, so that it can appear in interpolants computed in later iterations.^5^

Soundness of Algorithm 1 as stated in the following proposition can be proved by a straightforward induction on the quantifier prefix using Theorem 2 and Proposition 1.

#### Proposition 2

Given a quantified Boolean formula $\varPhi$ and a quantifier type $Q \in \{\forall , \exists \}$, Algorithm 1 terminates with a (possibly empty) set $\{\,(x_1, I_1) \dots (x_k, I_k) \,\}$ of pairs $(x_i, I_i)$ such that $I_i$ represents the unique strategy function of $x_i$ in $\varPhi$ and $var (x_i) \in var _Q(\varPhi )$ for $1 \le i \le k$.


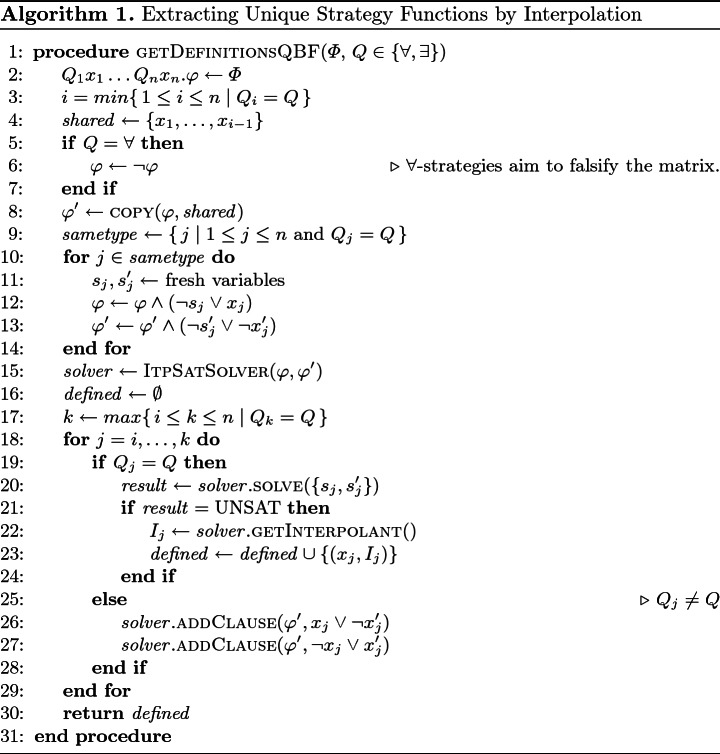


#### Example 2

Consider the QBF $\varPsi = \forall x_1 \exists y_1 \forall x_2 \exists y_2. \varphi$, where

$$\begin{aligned} \varphi = (x_1 \vee y_1) \wedge (\lnot x_1 \vee \lnot y_1) \wedge (x_2 \vee y_2) \wedge (\lnot x_2 \vee \lnot y_2). \end{aligned}$$

We illustrate a run of Algorithm 1 on $\varPsi$ with $Q = \exists$. Since $y_1$ is the leftmost existential variable, we create a copy $\varphi '$ of $\varphi$ with every variable except $x_1$ renamed, that is,

$$\begin{aligned} \varphi ' = (x_1 \vee y_1') \wedge (\lnot x_1 \vee \lnot y_1') \wedge (x_2' \vee y_2') \wedge (\lnot x_2' \vee \lnot y_2'). \end{aligned}$$

We also add the clauses $(\lnot s_1 \vee y_1)$ and $(\lnot s_2 \vee y_2)$ to $\varphi$ and the clauses $(\lnot s_1' \vee \lnot y_1')$ and $(\lnot s_2' \vee \lnot y_2')$ to $\varphi '$. In the main loop, Algorithm 1 first checks whether $\varphi \wedge ~\varphi '$ is unsatisfiable under the assumptions $\{s_1, s_1'\}$. Unit propagation simplifies $\varphi$ to (omitting unused selector variables and clauses)

$$\begin{aligned} (\lnot x_1) \wedge (x_2 \vee y_2) \wedge (\lnot x_2 \vee \lnot y_2), \end{aligned}$$

and $\varphi '$ simplifies to

$$\begin{aligned} (x_1) \wedge (\lnot x_2' \vee y_2') \wedge (\lnot x_2' \vee \lnot y_2'). \end{aligned}$$

By resolving $(\lnot x_1)$ with $(x_1)$ we obtain the empty clause, and $\lnot x_1$ is the corresponding interpolant,^6^ so $(y_1, \lnot x_1)$ is added to the set of definitions. Next, we consider the universally quantified variable $x_2$ and add the clauses $(x_2 \vee \lnot x_2')$ and $(\lnot x_2 \vee x_2')$ to $\varphi '$. Finally, we check whether $y_2$ is definable by calling the SAT solver under the assumptions $\{s_2, s_2'\}$. Now, the formula $\varphi$ simplifies to

$$\begin{aligned} (x_1 \vee y_1) \wedge (\lnot x_1 \vee \lnot y_1) \wedge (\lnot x_2), \end{aligned}$$

and $\varphi '$ simplifies to

$$\begin{aligned}&(x_1 \vee y_1') \wedge (\lnot x_1 \vee \lnot y_1')\, \wedge \\ {}&(x_2') \wedge (x_2 \vee \lnot x_2') \wedge (\lnot x_2 \vee x_2'). \end{aligned}$$

Unit propagation derives the clause $(x_2)$ from the clauses in the second line, which can be resolved with the clause $(\lnot x_2)$ from $\varphi$ to obtain a resolution refutation of the formula $\varphi \wedge \varphi '$, with $\lnot x_2$ as an interpolant. Accordingly, $(y_2, \lnot x_2)$ is added to the set of definitions. Algorithm 1 terminates with the definitions $\{(y_1, \lnot x_1), (y_2, \lnot x_2)\}$, and it is readily verified that $y_1 \equiv \lnot x_1$, $y_2 \equiv \lnot x_2$ is indeed the unique existential winning strategy of $\varPsi$.

### Improvements and Generalization to Dependency QBF

Consider a QBF $\varPhi = \forall x_1, x_2\, \exists y_1, y_2 . (x_1 \leftrightarrow x_2) \leftrightarrow (y_1 \leftrightarrow y_2)$. It is easy to verify that $\varPhi$ is true and that $y_1$ and $y_2$ do not have unique Skolem functions: for every assignment to the universal variables there are two ways of setting $y_1$ and $y_2$ so as to satisfy the matrix, so neither existential variable is defined by the universal variables alone. However, each variable is defined by all remaining variables. For instance, variable $y_2$ is defined by $x_1, x_2$, and $y_1$.

More generally, increasing the set of defining variables allows us to detect more definitions: if *x* is defined in terms of *X* then it is also defined in terms of any enclosing set $X' \supset X$. To exploit this, we modified Algorithm 1 so as to assume a total ordering of variables and check for definitions of a variable *x* in terms of *all* variables *X* which precede it in the quantifier prefix. This can be implemented by simply adding clauses encoding equivalence of $x_j$ and $x_j'$ (lines 26–27) regardless of quantifier type.

Technically, this leads to an alternative definition of a “winning strategy” for a QBF where each strategy function takes an assignment to all preceding variables as input. Both definitions are ultimately equivalent in the sense that a winning strategy according to one definition can be transformed into a winning strategy according to the other definition without changing its responses (cf. the work on quantifier elimination by functional composition and self-substitution [8, 14, 28, 29]). One can prove an analogue of Proposition 1 stating that the strategy function—according to the alternative definition—of a variable *x* is unique whenever *x* is defined in terms of the variables preceding *x* in the quantifier prefix.

*Dependency Quantified Boolean Formulas (DQBFs)* generalize QBFs by allowing a non-linear quantifier prefix. More specifically, each existential variable is annotated with a set of universal variables its Skolem function may depend on. A DQBF is true if there is an existential winning strategy such that each Skolem function satisfies these restrictions [2]. Although evaluating DQBF is NEXPTIME-complete and thus believed to be much harder than evaluating QBF, the fact that problems can be concisely encoded in DQBF [12, 18] has prompted the development of dedicated DQBF solvers [13, 15, 17, 48].

Algorithm 1 can easily be extended to compute unique Skolem functions of DQBF. The standard DQDIMACS format [15] allows for the combination of a linear quantifier prefix with variables for which the dependency sets are explicitly stated. The linear quantifier prefix can be handled as before. For each existential variable *x* with explicit dependency set $D_x$ we simply check whether *x* is defined by $D_x$. If multiple variables $x_1, \dots , x_k$ have the same dependency set $D_x$ (which is frequently the case in benchmark formulas) we check whether $x_i$ is defined by $D_x \cup \{x_1, \dots , x_{i-1}\}$ for each $1 \le i \le k$. Again, this technically requires a non-standard definition of Skolem functions for DQBF but can easily be proven sound.

## Implementation

We implemented the algorithm described in the previous section in a prototype named Unique. As a back end SAT solver we use ItpMiniSat, a modified version of MiniSat [11] bundled with the ExtAvy model checker that efficiently generates interpolants in memory and supports both assumptions and incremental solving [22, 49]. Unique can read PCNF formulas (QDIMACS), prenex non-CNF QBFs (QCIR), as well as DQBFs with CNF matrices (DQDIMACS).

Interpolants obtained from ItpMiniSat are represented as And-Inverter graphs (AIGs) and accessed through the AIG library of ABC [7]. To make use of the structural sharing capabilities of AIGs, we maintain a single AIG representing the interpolants computed in the main loop (lines 18–29) of Algorithm 1. Whenever a new interpolant is obtained, the corresponding AIG returned by ItpMiniSat is merged into the existing AIG. If the number of AIG nodes exceeds a (geometrically increasing) threshold, we use the ABC macro *compress2* to reduce the size of the combined AIG. Upon termination, and assuming the AIG is not too large, this is followed up by a round of *FRAIGing* [37] and a final application of *compress2*.

While running Unique on QBFs with multiple quantifier alternations we noticed that ItpMiniSat got stuck attempting to solve some of the definability queries. Further testing revealed that the corresponding instances were hard for most state-of-the-art solvers. Increasing the overall timeout would allow us to solve these instances in some cases, but naturally the corresponding interpolants (for unsatisfiable instances) were very large (and difficult to compress with ABC). This clearly defeats the purpose of detecting unique strategy functions quickly. We thus decided to impose a limit on the number of conflicts for each call of ItpMiniSAT (currently set to 1000 conflicts). This significantly reduces the overall running time of Unique for many instances and ensures that individual interpolants are small, but only marginally decreases the total number of definitions found.

Since the individual definability queries are independent of each other, it is not necessary to determine for each input variable whether it is defined. Accordingly, we implemented Unique as an *anytime* algorithm: upon termination, it returns the set of variables with unique strategy functions identified up to that point, along with the AIG representing the corresponding functions.

## Experiments

For the experiments described below we used a cluster with Intel Xeon E5649 processors at 2.53 GHz running 64-bit Linux.

### Gate Extraction

We first ran Unique to compute unique strategy functions for the instances in the 2QBF (402 instances) benchmark set from the 2018 QBF Evaluation, as well as the PCNF (558), QCIR (341), and DQBF (333) benchmark sets from the 2019 QBF Evaluation.^7^ For each job we imposed a time limit of 600 s and a memory limit of 1.8 GB.

**Fig. 1.**
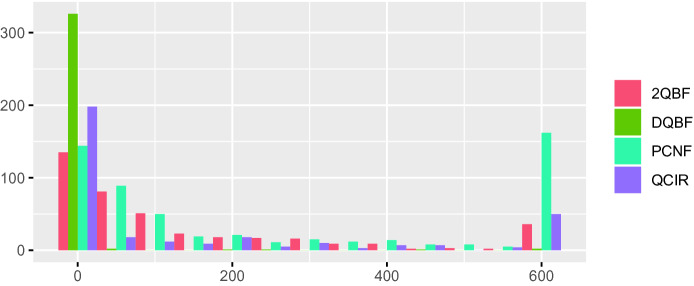
Running time (s) of Unique by benchmark set. For each 50-s interval within the time limit (x-axis), the number of instances (y-axis) processed by Unique with a running time in that interval is shown.

Figure 1 shows a histogram for the running time of Unique on different benchmark sets. While most instances are processed quickly, Unique runs into the time limit for a significant number of PCNF instances. Generally, the running time increases with the size of the matrix and the number of variables. This explains why almost all DQBF formulas are processed quickly, as these tend to be much smaller compared to formulas from the other benchmark sets.

**Fig. 2.**
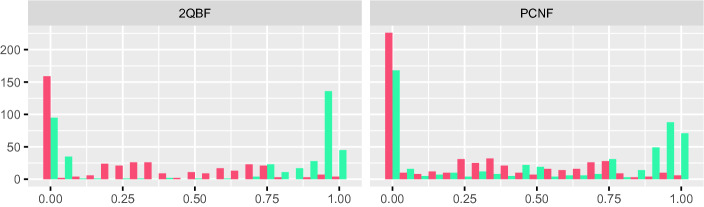
Fraction of existential variables with unique strategy functions in 2QBF (left) and PCNF (right) instances before (turquoise) and after (red) syntactic gate detection. For each fraction (x-axis) we see the number of instances (y-axis) with the corresponding fraction of unique existential strategy functions. (Color figure online)

Figure 2 shows a histogram for the fraction of existential variables with unique strategy functions in 2QBF and PCNF instances (turquoise bars). We clearly see a bimodal distribution here: there is a large number of instances where the strategy functions of most variables are unique, but also a significant number of instances where few existential strategy functions are unique. To determine how many of the corresponding definitions cannot be found by syntactic gate detection, we used the QCIR-conv script provided by GhostQ [31] to convert 2QBF and PCNF instances to QCIR, and ran Unique again on the resulting circuits. To do this, the circuit is translated (back) to CNF, but auxiliary variables representing gates are ignored by the definability check. Testing showed that a one-sided CNF encoding [42] works better than standard Tseitin conversion.

Table 1 (left) shows quartiles for the distributions of unique existential strategy functions detected by Unique in each benchmark set.^8^ We only show the distribution for existential variables in Table 1 and Fig. 2 since very few universal variables were found to have unique strategy functions. In fact, only 51 instances from the QCIR benchmark set encoding bounded synthesis for Petri games contained such universal variables.

**Table 1.** Distribution (quartiles) of the fraction of unique Skolem functions identified by Unique before (left) and after (right) preprocessing with HQSPre. Rows marked by a star (*) show the distribution after syntactic gate detection.

	Original			Preprocessed		
	1st	Median	3rd	1st	Median	3rd
2QBF	0.03	0.9	0.96	0	0	0
2QBF*	0	0.22	0.54	0	0	0
PCNF	0	0.53	0.94	0	0	0.03
PCNF*	0	0.21	0.53	0	0	0.02
QCIR	0	0	0.13	–	–	–
DQBF	0.57	0.88	0.94	0	0.22	0.45

The fraction of variables with unique strategy functions was smallest for QCIR instances. This is expected, since they can represent circuit structure directly and do not require auxiliary variables to encode gate definitions. By contrast, 2QBF and DQBF instances contain many variables with unique strategy functions. For about half of the instances, between roughly $90\%$ and $95\%$ of the existential strategy functions are unique.

On the right of Table 1 we show the distribution of unique existential strategy functions after preprocessing with HQSPre [52]. Clearly, only very few unique Skolem functions are detected by Unique. This may be in part due to the fact that preprocessing detects and removes gate definitions [27]. Another possibility is that definitions are simply lost: some of the most powerful preprocessing techniques for QBF currently used only preserve the truth value and not the set of strategies [23]. We will return to this topic at the end of the next subsection.

### Solving Formulas Augmented with Definitions

Unique strategy functions of a (D)QBF can be substituted for their variables without changing the set of winning strategies. This can be used in preprocessing to reduce the number of quantified variables, typically at the cost of increasing the size of the matrix. In the following experiments, we substituted definitions found by Unique for the defined variables and ran QBF and DQBF solvers on the resulting instances.

First, we considered the 2QBF benchmark set. We picked the QCIR solvers Quabs [47], QFun [26], and GhostQ [31], along with the dedicated 2QBF (PCNF) solver CADET [43]. For the QCIR solvers, the performance on instances constructed by syntactic gate detection with QCIR-conv serves as a baseline. We compare it with performance on instances obtained by Unique  and—since QCIR-conv also performs circuit-level simplifications that go beyond gate extraction—with a combination of both where QCIR-conv and Unique are run in sequence.

**Fig. 3.**
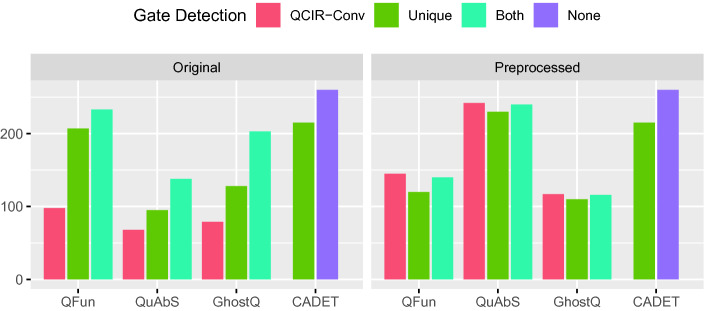
Number of 2QBF instances solved (y-axis) by solvers (x-axis) using different gate detection methods before (left) and after (right) preprocessing with HQSPre.

For CADET, we compare performance on the original 2QBF instances with performance on QDIMACS instances augmented with CNF encodings of definitions extracted by Unique. For each configuration, we report the number of instances solved within a time limit of 15 min. To isolate the effect of adding definitions, the time required by Unique (and QCIR-conv) is not counted towards the time limit.^9^ The results are shown in Fig. 3 (left).

QFun, Quabs, and GhostQ benefit considerably from semantic gate extraction, in particular when applied on top of syntactic gate extraction. By contrast, CADET solves *fewer* instances augmented with gate definitions than original instances. We found this surprising, since variable definitions should be detected by CADET’s heuristic for identifying unique Skolem functions. Perhaps most definitions found by Unique are already covered in this way, so that the additional clauses simply slow down propagation. We believe that explicitly telling CADET which variables have already been identified as determined should result in a speedup overall.

**Fig. 4.**
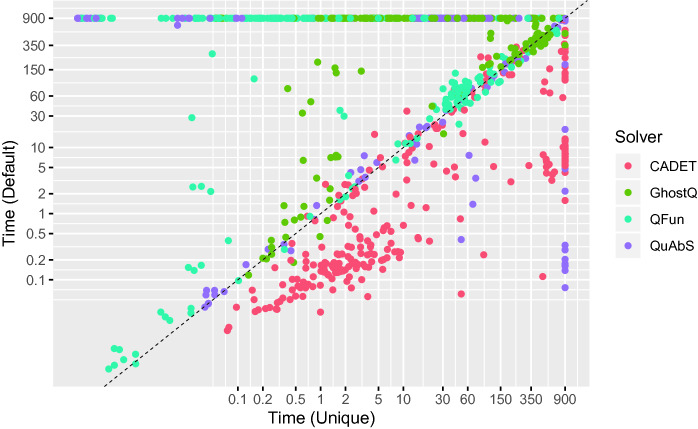
Solving time (s) for 2QBF instances with (x-axis) and without Unique (y-axis).

Figure 4 takes a closer look at solving times for individual instances (for this plot, memory outs are treated as timeouts). CADET is slower on instances augmented by Unique but fairly consistent, while the effect on the other solvers is more erratic. We conjecture that this is because the set of existential strategies is preserved and the instances thus “look similar” to CADET.

**Fig. 5.**
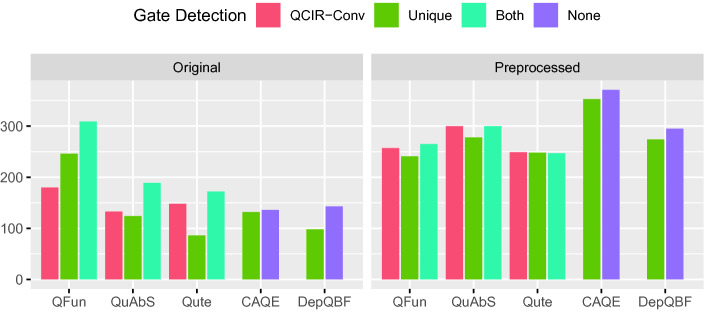
PCNF instances solved (y-axis) by solver (x-axis) using different methods for gate detection before (left) and after (right) preprocessing with HQSPre.

Next, we tested with PCNF instances and considered the QDIMACS solvers DepQBF [5] and CAQE [44], as well as the QCIR solvers Quabs [47], QFun [26], and Qute [40]. Again, we compare the number of instances solved in 15 min with different options for gate detection. Results are shown in Fig. 5 (left). Again all QCIR solvers benefit from gate detection with Unique when performed on top of syntactic gate detection with QCIR-Conv, while performance decreases for both QDIMACS solvers. The additional clauses and variables introduced by Unique apparently do not help these solvers and simply result in a slowdown.

Finally, we tested the impact of Unique on DQBF (DQDIMACS) instances solved by HQS [19] and DCAQE [48] within 15 min. Since DQBF solvers currently do not (yet) support non-CNF input, we translate definitions to CNF and add them to the original formulas. Note that whenever an existential variable *x* is defined by (a subset of) its dependency set, we can safely let *x* depend on additional variables. This is sound since the response of variable *x* is already determined by the variables in the original dependency set and cannot change depending on other inputs. In particular, we can collect all defined variables (and auxiliary variables) in an “innermost” existential quantifier block that depends on all universal variables. Since many existential variables have uniquely determined strategy functions (see Table 1), this allows us to push many variables into the innermost quantifier block and get closer to a linear quantifier prefix. For HQS, this translates into a small increase in the number of solved instances (208 vs. 189), whereas DCAQE basically solves the same number of instances (133 vs. 135).

*Interaction with Preprocessing.* QBF solvers for PCNF are typically paired with preprocessors such as Bloqqer [6] or HQSPre [52]. These are highly engineered tools that batter instances with a barrage of techniques and can often solve formulas completely on their own. Most solvers benefit greatly from preprocessing. This is evident in Fig. 5 (right), which shows the number of solved PCNF instances with different forms of gate detection after preprocessing with HQSPre (within a timeout of 600 s). Here, the number of solved instances increases significantly for almost all systems.

At the same time, preprocessing appears to obscure or destroy definitions. Unique hardly finds any definitions in preprocessed instances (cf. Table 1) and accordingly has little impact on performance. For QFun, which benefitted most from gate detection in our experiments, this translates to a substantial reduction in the number of solved instances. On the 2QBF benchmark set (Fig. 3), both QFun and GhostQ solve significantly fewer instances with HQSPre compared to the combination of Unique and QCIR-Conv, whereas the number of solved instances almost doubles for QuAbS. Understanding which preprocessing techniques obscure gate definitions and why certain solvers benefit more from gate detection than others are important questions for future work.^10^

## Related Work

Our semantic gate detection technique is closely related to a method for *determinizing* Boolean relations by Jiang et al. [29], a problem that essentially corresponds to solving 2QBF. The authors show that, for a (total) relation *R*(*X*, *y*) with a single output variable *y*, a functional implementation of *y* can be obtained as an interpolant for $\lnot R(X,0) \wedge \lnot R(X,1)$. This can be used to determinize relations *R*(*X*, *Y*) with a set of output variables $Y = \{y_1, \dots , y_n\}$. First, an implementation $f_n$ for $y_n$ can be computed by treating *R* as a relation with inputs $X \cup \{y_1, \dots , y_{n-1}\}$ and single output $y_n$. Subsequently, the implementation $f_n$ can be substituted for $y_n$ to obtain a relation $R'(X, Y \setminus \{y_n\})$. By repeating this process, a functional implementation $f_1$ of $y_1$ can eventually be obtained. Substituting $f_i$ into $f_{i+1}$ for $1 \le i < n$ results in functional implementations that only depend on the original input variables *X*. This approach does not require for any of the output variables to be defined by *X*, but an implementation of $y_i$ solely in terms of the input variables *X* is only available at the very end of this process. For *deterministic* relations *R*(*X*, *Y*) (where every *y* is defined in terms of *X*), the authors show that a functional implementation of $y \in Y$ can be obtained as the interpolant of a formula that corresponds to the formula in the statement of Padoa’s theorem. Our result stated as Theorem 2 is more general in that it holds for multi-output relations that are not necessarily deterministic.

Hofferek et al. use interpolation to synthesize multiple functional implementations from a single proof and thus avoid the increase in formula size incurred by repeated substitution [24]. This has an analogue in *strategy extraction* for QBF, which allows for implementations of all (existential or universal) variables to be obtained from a proof [3]. However, strategy extraction requires the input QBF has been solved, whereas our main interest is in preprocessing QBF.

There is a series of works on recovering gate definitions from CNF formulas. Li integrated rules for detecting equivalent literals in a Davis-Putnam style algorithm [35]. Ostrowski et al. represent formulas as graphs to detect patterns corresponding to and-gates, or-gates, and equivalences [38]. Roy et al. use CNF signatures to detect a richer set of gates [45]. Fu and Malik extend this to arbitrary (user-specified) gate libraries and ensure that a maximum acyclic circuit is constructed [16].

In the context of QBF, Bacchus and Goultiaeva showed that circuit reconstruction can speed up solvers by providing them with a better set of initial cubes [21]. They also extended the scope of these techniques to CNF formulas obtained from circuits by the Plaisted-Greenbaum encoding [42]. Scholl and Pigorsch developed a QBF solver that manipulates an AIG representation of the matrix to perform quantifier elimination and relies on circuit reconstruction to simplify the initial AIG [41].

Balabanov et al. proposed a SAT-based semantic gate extraction technique [4]. Their approach has the disadvantage that a subset of clauses inducing a definition has to be guessed. As a more efficient heuristic, they suggest to identify *pseudo* definitions instead. A set of clauses $(A_1 \vee x),\dots ,(A_k \vee x),(B_1 \vee \lnot x),\dots ,(B_l \vee \lnot x)$ is a pseudo definition of *x* if the formula $A_1 \wedge \dots \wedge A_k \wedge B_1 \wedge \dots \wedge B_l$ is unsatisfiable. Rabe and Seshia use a similar criterion in their *incremental determinization* algorithm to identify variables that are *(locally) deterministic* [43]. Checking for pseudo definitions is typically efficient but limits the range of definitions that can be detected.

## Conclusion

Syntactic gate detection has been shown to benefit SAT solvers [10, 16] and QBF solvers [21]. The underlying algorithms are fast but limited to a predefined library of gates. By contrast, our semantic gate extraction method can detect any definition entailed by an input formula but requires an interpolating SAT solver. In the context of SAT, this overhead likely outweighs any potential benefits. However—as demonstrated by our experiments—there is significant potential for application to harder problems such as QBF and DQBF evaluation. Here, preprocessing is just a first step.

At the same time, our results show that substituting unique strategy functions can slow down solvers. In some sense, this is counter-intuitive: ideally, providing solvers with unique strategy functions should give them a head start, or at least not hurt their performance. By analogy, if we give a SAT solver part of a backbone assignment, it can simply instantiate accordingly and need not consider the corresponding variables for the remainder of its run. With the exception of CADET, QBF solvers currently cannot “instantiate” variables with strategy functions in this way, since they are only equipped to reason about assignments. We believe that designing techniques for reasoning about *strategies* is a key challenge in developing the next generation of QBF solvers.

## Footnotes

1 We sometimes refer to existential strategy functions as *Skolem* functions and universal strategy functions as *Herbrand* functions.

2 Assuming the SAT solver does not use the full power of the DRAT proof system [51].

3 In fact, the result holds even for first order logic, but we will confine ourselves to the propositional case.

4 Two distinct selector variables are required to ensure that they do not belong to the common variables of $\varphi$ and $\varphi '$.

5 One could also add these clauses to $\varphi$, in which case $x_j'$ would become part of the shared vocabulary. This has the slight disadvantage that subsequently computed definitions may use a mixture of variables from $\varphi$ and $\varphi '$, rather than just $\varphi$.

6 As mentioned above, interpolants can be efficiently extracted from resolution refutations [32, 36, 46].

7 http://www.qbflib.org.

8 For instance, the left side of the first row of Table 1 says that for $75\%$ of 2QBF instances, Unique was able to identify 3% of Skolem functions as unique; for half of the instances, at least 90% of existential variables were identified as having unique Skolem functions; and for 25% of instances, at least 96%.

9 The results are qualitatively the same when the running time of Unique is counted towards the time limit: the largest decrease in the number of solved instances across all benchmark sets and configurations is 7.

10 We also ran experiments with QCIR-conv and Unique applied *before* preprocessing. The results were significantly worse, so we do not report them in detail. Standard preprocessing requires PCNF input, so that definitions have to be encoded using additional clauses and Tseitin variables. Just like the PCNF solvers in the other experiments, HQSPre appears to be unable to do anything useful with these extra clauses and variables.
